# Anthropometric Characteristics and Weight Status of Early Adolescents (Aged 12–14) in Montenegro; Urban–Rural and Regional Differences

**DOI:** 10.3390/children10101664

**Published:** 2023-10-08

**Authors:** Borko Katanic, Dusko Bjelica, Mima Stankovic, Zoran Milosevic, Jovan Vukovic, Amel Mekic

**Affiliations:** 1 Faculty of Sport and Physical Education, University of Nis, 18000 Nis, Serbia; borkok@ucg.ac.me; 2 Faculty for Sports and Physical Education, University of Montenegro, 81400 Niksic, Montenegro; dbjelica@ucg.ac.me; 3 Faculty of Sport and Physical Education, University of Novi Sad, 21000 Novi Sad, Serbia; zoran.milosevic@uns.ac.rs (Z.M.); jovanvukovic@uns.rs (J.V.); 4 Faculty of Sports and Physical Education, University of Sarajevo, 71000 Sarajevo, Bosnia and Herzegovina; amel.mekic@fasto.unsa.ba

**Keywords:** morphological characteristics, BMI status, prevalence of obesity, pupils, type of settlement category, residential status

## Abstract

The aim of this study was to determine urban–rural and regional differences in anthropometric characteristics among adolescents aged 12–14, as well as to present the prevalence of weight status. A total of 534 adolescents aged 12–14 from primary schools across Montenegro participated in this cross-sectional study (283 boys, aged 13.52 ± 0.42, body height 169.43 ± 8.89, body weight 60.54 ± 13.47; 251 girls, aged 13.51 ± 0.40, body height 165.54 ± 6.67, body weight 55.28 ± 9.27). The sample was divided by geographic region in Montenegro into northern, central, and coastal regions, and according to settlement type into urban and rural inhabitants. Anthropometric characteristics were assessed using a battery of seven variables: arm span (AS); body height (BH); body weight (BW); waist circumference (WC); hip circumference (HC); body mass index (BMI); and waist-to-hip ratio (WHR). BMI was categorized based on the World Health Organization’s (WHO) cut-offs. The results indicate that girls from urban areas exhibited significantly greater body height and lower BMI values compared to their rural counterparts. Similarly, boys from urban areas also demonstrated lower BMI values compared to their rural peers. However, no statistically significant differences were observed in other anthropometric characteristics between these two groups of adolescents. Based on analysis of variance (ANOVA) and post-hoc analysis, it was found that girls from central areas had significantly greater body height compared to those from northern and coastal areas. Likewise, boys from northern areas showed higher body mass and BMI values compared to those from central areas. Additionally, both boys and girls from central areas had higher values compared to those from coastal areas. Nevertheless, no significant differences were detected in other anthropometric characteristics among adolescents from these regions. This study identified significant differences in anthropometric parameters among participants based on urban–rural status and within regional divisions. However, further research encompassing a larger sub-sample and a broader array of anthropometric variables is needed to draw a more comprehensive conclusion.

## 1. Introduction

Children play a significant role in our society, and their development has been acknowledged as a key indicator for assessing population health trends and creating successful strategies [1]. All organisms go through several periods of growth, including morphological, psychological, mental, and social phases [2]. Adolescence encompasses the age range from 10 to 19 years and stands out as a rapid and crucial stage of human growth, encompassing physical, emotional, cognitive, and social aspects, among others [3]. Due to this significance, it becomes imperative to prioritize the well-being of adolescents by attending to all factors that can impact their health during this period.

Recent studies conducted on a substantial cohort of youngsters and adolescents have reported a noticeable upward trajectory in the prevalence of excess weight and obesity during the last twenty years [4,5,6]. Based on statistics from the World Health Organization (WHO), the occurrence of overweight and obesity among youngsters and teenagers has experienced a substantial increase, climbing from 4% in 1975 to more than 18% in 2016. Presently, it is estimated that there are approximately 340 million obese children and adolescents aged from 5 to 19 years globally [7]. Additionally, research conducted by Spiota and Luma [8] indicates that nearly one-third (31%) of children and adolescents worldwide aged 6 to 19 have excess body weight compared to normal values.

For these reasons, there is a need for monitoring the physical development of children, and one of the most reliable methods to define the human body and track how physical growth processes change with age, particularly during adolescence, is through anthropometric characteristics and body composition. Accordingly, it is possible to predict what adolescence would look like at a given age and help determine the potential health concerns that may come along with it in later life [9]. Because of its affordability, ease of application, and non-intrusive characteristics, the utilization of anthropometric screening for assessing cardiometabolic risk in youngsters and teenagers has gained significant popularity. Additionally, body mass index (BMI) readings over 20–25 kg/m^2^ have been linked to an estimated five million fatalities [10]. There are other aspects to researching anthropometric characteristics and weight status during adolescence. During this phase of life, adolescents often lack an understanding of how to cope with the natural changes their bodies undergo, a concept referred to as “body perception”. Alongside psychological, behavioral, and social shifts, these changes might contribute to adolescents developing a negative body image and feeling dissatisfied with their body’s form and structure [11]. Consequently, adolescents might adopt certain behaviors that are seemingly beneficial, such as engaging in exercise, vigorous physical activities, or going to the gym for bodybuilding. On the other hand, they might also adopt unhealthy behaviors like avoiding physical activities altogether, consuming unhealthy foods, being inactive and unenergetic during leisure time, indulging in video games, staying up late, and sleeping irregularly. These actions could disrupt the normal processes of growth during this stage and potentially lead to health issues [12].

A geographic region categorized as urbanized, based on its settlement characteristics, stands out due to its higher population density and extensive human-made infrastructure, in contrast to the surrounding regions. Conversely, a rural area is identifiable by its lower population density and a larger proportion of land dedicated to agricultural activities compared to the neighboring areas [13]. Research has revealed that various aspects of health and weight status vary based on socioeconomic status and geographic region [14]. Hence, having epidemiological data specific to each country regarding the prevalence of anthropometric traits, body composition, and components of health-related fitness among adolescents is crucial. These data are essential for crafting effective public health strategies and creating appropriate interventions for promoting physical activity [15].

Numerous studies have investigated the variations in anthropometric measures and weight status among adolescents across different countries and areas [1,13,16,17]. In a study conducted by Tishukaj et al. [13], the focus was on examining physical fitness and anthropometric traits among adolescents residing in either urban or rural areas of Kosovo. The study found a significant occurrence of overweight and obesity, particularly among boys aged from 14 to 15, and this prevalence was consistent across both rural and urban locations. On the other hand, Choudhary et al. [16] found that the average height was notably greater among urban girls. In addition, the average body mass index (BMI) of adolescents was significantly higher in rural regions compared to their urban counterparts. However, there is a noticeable lack of research that specifically differentiates between urban and rural settings in Montenegro.

Moderate levels of physical activity are linked to cardiovascular health, and positive changes in anthropometric characteristics, weight status, and excessive or intense activity levels during childhood and adolescence could hinder healthy physical growth and development. Prior research had not investigated the anthropometric characteristics and weight status of early adolescents within Montenegrin society. Therefore, the objective of this study was to assess disparities in urban–rural and regional anthropometric characteristics among 12 to 14-year-old adolescents and to provide insights into the prevalence of weight status.

## 2. Materials and Methods

### 2.1. Participants

The sample consisted of early adolescents aged 12–14 from primary schools across Montenegro. A total of 534 pupils participated in this cross-sectional study (283 boys, aged 13.52 ± 0.42, body height 169.43 ± 8.89, body weight 60.54 ± 13.47; 251 girls, aged 13.51 ± 0.40, body height 165.54 ± 6.67, body weight 55.28 ± 9.27). The criteria for the inclusion and selection of subjects were as follows: healthy children (children without any diseases or disorders) of both genders, aged 12 to 14 years, who were students in an elementary school in Montenegro and were not participating in any sports program. Exclusion criteria applied to children younger or older than the specified age range, children with specific developmental disabilities and various illnesses, and children who were engaged in specific sports activities. The sample was stratified based on gender, geographic location, and settlement type. Geographic categorization in Montenegro was determined according to the northern, central, and coastal regions [18]. Settlement types were classified using administrative criteria and population size, where rural areas encompassed villages and small towns with populations of 10,000 or fewer residents, while urban areas comprised locations with over 10,000 inhabitants [19]. Table 1 provides detailed characteristics of the study population. The participation of students was voluntary, and the research process adhered to the principles outlined in the Helsinki Declaration, with obtained written consent from parents. This study was approved by the Ethics Committee of the Faculty of Sport and Physical Education, University of Novi Sad (decision number: 49-02-04/2023-1; date: 27 February 2023).

### 2.2. Measurements

The testing of all children in this cross-sectional study was conducted from 5 May to 15 May 2023. The standard international biological procedure was used to determine the morphological characteristics [20]. Anthropometric characteristics were assessed using a battery of seven variables: arm span (AS); body height (BH); body weight (BW); waist circumference (WC); and hip circumference (HC). Body mass index (BMI) was calculated based on the standard formula: BMI = BM (kg)/BH (m)^2^ (BM—body mass, BH—body height), while waist-to-hip ratio (WHR) was calculated by dividing WC (in cm) by hip circumference (cm). The categorization of BMI followed the World Health Organization’s (WHO) guidelines, classifying individuals as underweight, normal weight, overweight, or obese [21]. BMI has a high correlation with body fat content, making it a valuable indicator of nutritional status in children [22].

### 2.3. Statistics

Basic parameters of descriptive statistics were calculated: arithmetic mean; standard deviation; minimum; maximum; and percentages. Pearson correlation between anthropometric characteristics was used. The strength of the correlation was determined according to Cohen [23], where a weak correlation was defined as r = 0.1–0.29, a moderate correlation as r = 0.3–0.49, and a strong correlation as r = 0.5–1.0. To determine differences in anthropometric characteristics between Urban and Rural groups, a *t*-test for small independent samples was used, and to determine differences according to geographic region, a One-factor ANOVA was used. In all statistical analyses, significance was considered when *p* < 0.05. Data processing was carried out using the statistical software SPSS 26 (Statistical Package for Social Sciences, v26.0, SPSS Inc., Chicago, IL, USA) and Microsoft Excel (Microsoft Corporation, Redmond, WA, USA, version 13).

## 3. Results

Based on Table 1, the total sample size of participants was 534. Out of the total number of individuals, 53% were boys, and 47% were girls. The highest proportion was from the central region (71.54%). The urban-to-rural ratio of participants was 71.16% to 28.84%.

Table 2 provides descriptive statistics. The average age for boys was 13.52 ± 0.42; for girls, it was 13.51 ± 0.40, and the combined average for both groups was 13.52 ± 0.41 years. Boys had a higher body height and mass (169.43 ± 8.89 cm and 60.54 ± 13.47 kg, respectively) compared to girls (165.54 ± 6.67 cm and 55.28 ± 9.27 kg). The average BMI for boys was 20.99 ± 3.78, and for girls, it was 20.13 ± 2.99. The waist-to-hip ratio (WHR) for boys was higher (0.85 ± 0.06) compared to girls (0.81 ± 0.08).

Table 3 displays the correlations among various anthropometric characteristics within the studied group. High correlations were observed between similar measurements such as body height and arm span (0.839), body mass and BMI (0.876), waist circumference and body mass (0.740), waist circumference and BMI (0.674), waist circumference and hip circumference (0.737), hip circumference and body mass (0.697), and hip circumference and BMI (0.620). The waist-to-hip ratio did not exhibit significant correlations with most other measurements. The values between the waist-to-hip ratio and other measurements (0.052, 0.028, 0.144, 0.151) suggested that these parameters were less strongly associated with WHR.

**Figure 1:**
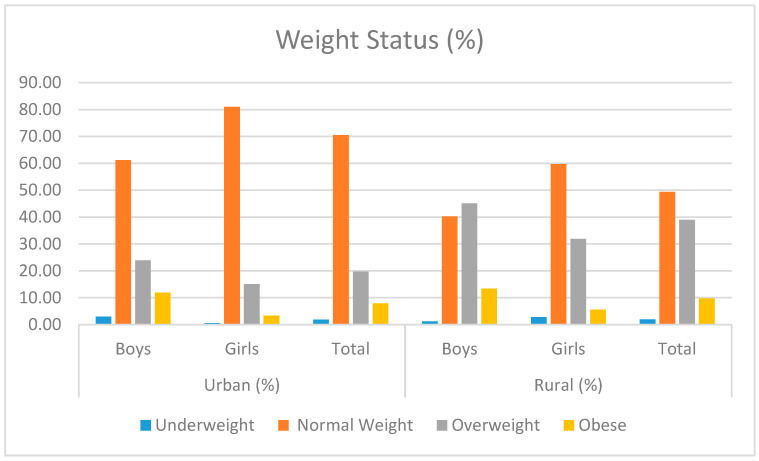
Prevalence of underweight, normal weight, overweight, and obesity in adolescents by type of settlement.

In Figure 1, the distribution of nutrition based on the participants’ weight status is shown. Among the urban adolescent population, 70.53% were within the normal weight range, and nearly 28% of adolescents were either overweight or obese (19.74% overweight and 7.89% obese). However, in the rural setting, this distribution differed, with 49.35% being normal weight and approximately 49% of participants being overweight or obese (38.96% overweight and 9.74% obese), with the highest percentage of obesity observed among male adolescents. The undernourished category was minimal, comprising only about 2%.

**Figure 2:**
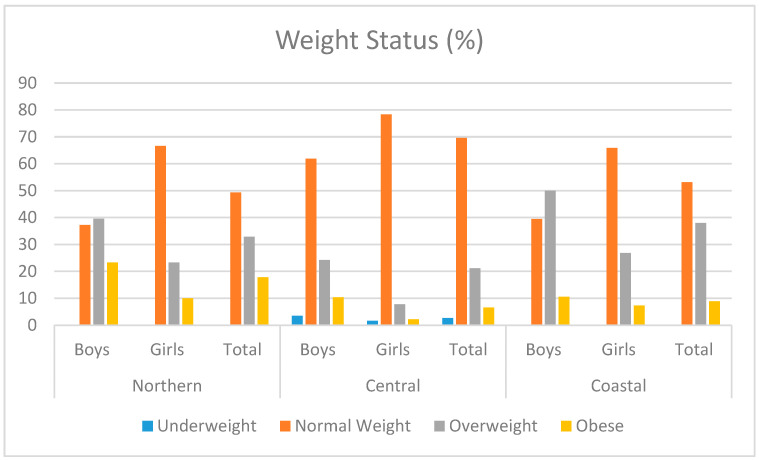
Prevalence of underweight, normal weight, overweight, and obesity in adolescents by geographic region in Montenegro.

Figure 2 offers an overview of the distribution concerning the participants’ geographic region. Adolescents from the northern region were 49.32% within the normal weight range, 32.88% overweight, and 17.81% obese. In the central region, these proportions were as follows: 69.63% normal weight; 21.15% overweight; and 6.54% obese. Meanwhile, in the coastal region, 53.16% were of normal weight; 37.97% were overweight, and 8.86% were obese.

Significant disparities were evident, as presented in Table 4, when comparing adolescents from urban and rural areas in terms of body height (*p* = 0.000) and body mass index (BMI) (*p* = 0.001). Girls residing in urban areas showed greater body height and lower BMI compared to their rural counterparts. Likewise, boys living in urban areas also displayed lower BMI values than their rural counterparts (*p* = 0.003). However, there were no significant differences observed in other anthropometric characteristics between these two groups of adolescents.

Significant differences in certain physical characteristics were identified based on ANOVA and post-hoc analysis, as is detailed in Table 5, among adolescents hailing from the northern, central, and coastal regions. Specifically, girls’ body heights showed significant differences among groups (*p* = 0.009), with girls from central areas having greater height compared to those from the northern and coastal areas. Additionally, body mass in boys and BMI in both genders showed significant differences (*p* = 0.016 for boys’ body mass, *p* = 0.000 for boys’ BMI, and *p* = 0.011 for girls’ BMI), with boys from northern areas having greater body mass and BMI compared to central areas. Moreover, boys and girls from different regions displayed statistically significant differences in waist-to-hip ratio (WHR) (*p* = 0.003 for boys and *p* = 0.007 for girls), with central areas showing higher values compared to coastal areas. Other characteristics, including arm span, waist circumference, hip circumference, and waist-to-hip ratio, did not exhibit statistically significant differences among children from these regions.

## 4. Discussion

This study confirmed high intercorrelations between anthropometric characteristics related to weight status: BM; BMI; WC; and HC. These findings align with numerous studies indicating that these parameters are strongly correlated and serve as robust predictors of obesity [24,25]. Furthermore, a significant correlation was also established between longitudinal dimensions such as BH and AS, which was consistent with previous research conducted on adolescent samples [26]. It is worth noting that these longitudinal parameters exhibited weak to moderate correlations with weight-related parameters, with the only strong correlation observed between BH and AS with BMI, consistent with prior research findings [27].

When examining the prevalence of obesity based on BMI, this study unveils that roughly 28% of children residing in urban areas fall into the overweight or obese category. In contrast, this percentage is notably higher in rural areas, reaching approximately 49% for children who are classified as overweight or obese. Data from other studies vary across different European countries, with Germany reporting an obesity prevalence of 8.7% [28], Finland of 13%, the Czech Republic of 16%, Greece of 33%, and Italy of 36% [29]. A recent study in Turkey indicates that this percentage is 30% for overweight and obese individuals [30]. Additionally, the findings indicate that the prevalence of overweight and obesity was higher in boys than in girls [13,31,32]. Based on these findings, it is evident that the percentages of both overweight and obese children in both urban and rural areas of Montenegro are highly concerning.

Looking at a broader range of anthropometric measures, significant differences between rural and urban participants were observed in terms of BH and BMI. Urban girls had higher BH (0.000) and lower BMI (0.001) compared to their rural counterparts, while urban boys had a lower BMI (0.003) than rural boys. Nonetheless, no significant differences were observed between adolescents from urban and rural areas in relation to other anthropometric parameters. It is worth noting that previous studies have generated varied findings, with some reporting no disparities in anthropometric characteristics among urban and rural adolescents [13,33,34,35]. Conversely, some studies have found that children from rural areas had lower BMI and WC values [36], BM and BMI [37], and other anthropometric characteristics [38], which contrasts with our study. The reasons for these discrepancies may be attributed to genetic factors, dietary habits, and the availability of physical activities in urban versus rural environments [37,39].

When analyzed regionally, the northern area exhibited the highest prevalence of overweight and obesity, at nearly 50%, followed by the coastal region with 39%, while the central region reported 28%. A comprehensive study [40] conducted in Montenegro during 2012/13, involving a sample of 4097 children aged 7–13 years (average age 10.2 ± 1.7), classified according to WHO standards, revealed a 27% prevalence of overweight and obese children in the country at that time. This result aligns with the central region but is considerably lower than the findings in the coastal and northern regions. A neighboring country reported a much higher obesity rate, with authors finding that 42% of children were overweight or obese in a sample of 322 participants [41]. Although these studies focused on a slightly younger age group (from 10 to 12 years old), we have considered these results due to the lack of studies with the appropriate age group in this region. However, it is essential to interpret these findings cautiously because, unlike the central region, which had a large sub-sample, the northern and coastal regions included relatively small sub-samples of children (*n* = 73 and 79).

Based on anthropometrics, ANOVA and post-hoc tests revealed significant differences among regions. Central region girls had significantly higher BH compared to girls from the northern region (0.009). Conversely, boys from the northern region had higher BM (0.016) and BMI (0.000) compared to boys from the central region. Boys from the central region also had a significantly higher WHR than boys from the coastal region (0.003), and central region girls had significantly higher values compared to girls from the northern and southern regions (0.007). No significant disparities were noted in other parameters. The only study that explored differences in the prevalence of adolescent obesity among these three regions in Montenegro [42] found no variation in obesity prevalence based on regional divisions, even though our study identified certain differences. The variation in findings could be attributed to the older age group included in the mentioned study and the fact that the authors exclusively evaluated distinctions using BMI, whereas our study utilized a more comprehensive set of anthropometric measurements. Despite the limited literature on the geographic regional impact in Montenegro, it is known that differences in the societal structure of a particular area, influenced by socioeconomic, infrastructural, cultural, and educational factors, variably affect adolescents’ participation in physical activities [43,44,45,46]. This has been confirmed by the impact of residential status, as urban-area children have greater opportunities to participate in physical activities through specialized sports programs compared to their rural counterparts [37,39]. In conclusion, these findings underscore the necessity for additional research in this domain to grasp the influence of geographic factors on adolescent nutrition.

This study has provided insights into the anthropometric and weight status of adolescents, underscoring specific distinctions tied to their residential status and internal regional divisions. This research confirms that obesity is becoming increasingly prevalent among children and adolescents and, according to the authors [47], is currently one of the most common public health problems. The high rate of overweight and obesity is concerning, especially when considering that obesity is associated with many health issues such as type 2 diabetes, asthma, hypertension, psychosocial problems, early atherosclerosis, etc. [48,49,50]. Therefore, the authors [51] warn that younger generations may live less healthy and shorter lives compared to their parents.

There are numerous potential reasons that have led to this situation. It is now considered that the main factors contributing to obesity are a sedentary lifestyle, insufficient physical activity (PA), and improper nutrition [52,53]. Colley et al. [54] found that children today spend as much as half of their waking time in a sedentary position, which reduces their time for PA. Numerous studies have established that obese children have lower levels of PA [55] and poorer motor skills compared to normal-weight children [56,57]. Therefore, promoting PA in the adolescent population could increase PA levels, contributing to a reduction in obesity rates.

Regarding residential conditions, it should be noted that the occurrence of obesity, in addition to endogenous (internal) factors, also depends on exogenous (external) factors such as geographic, climatic, and social factors [58]. A recent systematic review [59] indicates that climate change and global warming affect obesity rates in European countries. On the other hand, socioeconomic factors play a significant role, manifested through the different social structures of urban and rural areas, resulting in distinct lifestyle habits and ways of spending both school and leisure time. These factors also have varying effects on the physical activity of adolescents [43,44,45,46]. Additionally, the role of parents is crucial, especially since it is known that the physical inactivity of parents strongly influences children’s inactivity [60,61]. Therefore, there should be an appeal for parents to set an example by being more physically active themselves to encourage their children to adopt a more active lifestyle.

Certainly, monitoring anthropometric characteristics represents the first step in the prevention and treatment of obesity [62], and that is why anthropometry is important during adolescence to prevent health risks [63]. It is important to note that the World Health Organization (WHO), after comprehensive research on childhood obesity and the identification of significant issues, introduced the WHO Global Strategy on Diet, Physical Activity, and Health [7]. All of this supports the idea that childhood and adolescent obesity is a global phenomenon, and numerous world health organizations are striving to find solutions to this problem. However, it would be desirable for each country to follow these WHO guidelines by conducting comprehensive national research and, after accurately determining the situation, seek to implement a strategy within its borders. This emphasizes the significance of continuously monitoring the anthropometric and weight status of children and adolescents. These parameters serve as critical indicators of their quality of life and health during childhood and can also serve as predictors of health status throughout their lives [64,65].

This study represents a significant contribution to understanding the weight status of younger adolescents in Montenegro, as it is the first such research in the country to assess anthropometric status, covering the age range of 12–14 years in both genders and analyzing it according to their residential status and regional divisions within Montenegro itself. One of the key strengths of this study is the extensive sample, including a large number of adolescents in this age group. This allows for relevant and general conclusions to be drawn about the weight status of younger adolescents in Montenegro.

However, one limitation of this study is the small sub-sample size in regional divisions, and it may also be limited by the absence of parameters such as skinfold thickness or more precise measures of body composition that would provide a more detailed picture. Therefore, future research should include these additional measurement tools to obtain a more comprehensive analysis, and in addition, anthropometric and physical fitness tests should also be conducted. Conducting a large-scale national study on a substantial sample of participants, similar to studies conducted in other countries [28,40], would help overcome potential issues related to smaller sub-samples and provide a clearer insight into the state of the anthropometric status of adolescents in Montenegro.

## 5. Conclusions

This study reveals that approximately 28% of children in urban areas are classified as overweight or obese based on BMI, while this rate is significantly higher in rural areas, reaching around 49%. When examining a broader range of anthropometric measures, it was observed that urban girls had greater height and lower BMI compared to their rural counterparts, while urban boys had a lower BMI than rural boys. Looking at regional differences, the northern region has the highest prevalence of overweight and obesity, next to the coastal region and then the central region. Analysis of anthropometry and statistical tests uncover significant differences among regions, including height, body mass, BMI, and waist-to-hip ratio.

The information obtained from this research provides a foundation for the development of targeted interventions and policies aimed at improving the health of adolescents and preventing weight-related issues in this important population. This is in line with the guidelines of the group of authors [6] who emphasize the need to support healthy growth throughout the entire period from birth to adolescence through the healthcare system by enhancing the quality of nutrition, creating a healthier living environment, and providing high-quality preventive and curative care.

## Figures and Tables

**Table 1:** Characteristics of sample population (*n* = 534).

Gender		
Boys	283 (53.00%)	
Girls	251 (47.00%)	
Geographic region (number, %)		
Northern	73 (13.67%)	[♂ 43, ♀ 30]
Central	382 (71.54%)	[♂ 202, ♀ 180]
Coastal	79 (14.79%)	[♂ 38, ♀ 41]
Type of settlement (number, %)		
Urban	380 (71.16%)	[♂ 201, ♀ 179]
Rural	154 (28.84%)	[♂ 82, ♀ 72]

**Table 2:** Descriptive statistics.

		Mean	Std. Dev.	Min	Max
Age	Boys	13.52	0.42	12.2	16.0
	Girls	13.51	0.40	12.1	14.9
	Total	13.52	0.41	12.1	16.0
Arm Span (cm)	Boys	169.10	10.22	140.0	198.3
	Girls	164.19	7.45	141.0	185.0
	Total	166.79	9.34	140.0	198.3
Body Height (cm)	Boys	169.43	8.89	145.0	190.0
	Girls	165.54	6.67	151.0	187.0
	Total	167.60	8.15	145.0	190.0
Body Mass (kg)	Boys	60.54	13.47	34.6	110.2
	Girls	55.28	9.27	33.0	82.8
	Total	58.07	11.97	33.0	110.2
BMI	Boys	20.99	3.78	13.63	34.49
	Girls	20.13	2.99	13.91	32.49
	Total	20.58	3.45	13.63	34.49
Waist Circumference (cm)	Boys	73.74	9.23	52.0	114.5
	Girls	67.92	7.03	54.0	91.0
	Total	71.00	8.76	52.0	114.5
Hip Circumference (cm)	Boys	86.75	9.53	57.0	118.0
	Girls	84.16	8.91	62.0	117.0
	Total	85.53	9.32	57.0	118.0
WHR (Waist-to-Hip Ratio)	Boys	0.85	0.06	0.69	1.01
	Girls	0.81	0.08	0.65	1.24
	Total	0.83	0.07	0.65	1.24

**Table 3:** Correlation between anthropometric characteristics.

	Arm Span	Body Height	Body Mass	BMI	Waist Circumference	Hip Circumference
Body Height	0.839 **					
Body Mass	0.603 **	0.572 **				
BMI	0.242 **	0.120 **	0.876 **			
Waist Circumference	0.407 **	0.369 **	0.740 **	0.674 **		
Hip Circumference	0.399 **	0.382 **	0.697 **	0.620 **	0.737 **	
Waist-to-Hip Ratio	0.052	0.028	0.144 **	0.151 **	0.463 **	−0.223 **

** significant at the 0.01 level.

**Table 4:** Anthropometric characteristics of adolescents according to the type of settlement (*t*-test).

		Urban	Rural	*p*
Arm Span (cm)	Boys	169.26 ± 10.15	168.72 ± 10.44	0.690
	Girls	164.76 ± 7.52	162.77 ± 7.13	0.056
Body Height (cm)	Boys	169.95 ± 8.68	168.15 ± 9.33	0.123
	Girls	166.60 ± 6.53	162.91 ± 6.35	0.000 **
Body Mass (kg)	Boys	59.72 ± 13.56	62.54 ± 13.09	0.110
	Girls	54.84 ± 8.87	56.37 ± 10.19	0.265
BMI	Boys	20.56 ± 3.69	22.03 ± 3.82	0.003 **
	Girls	19.70 ± 2.65	21.19 ± 3.52	0.001 **
Waist Circumference (cm)	Boys	73.20 ± 9.24	75.06 ± 9.15	0.125
	Girls	67.96 ± 7.21	67.84 ± 6.64	0.898
Hip Circumference (cm)	Boys	86.05 ± 9.76	88.47 ± 8.73	0.053
	Girls	83.69 ± 9.14	85.33 ± 8.24	0.189
WHR (Waist-to-Hip Ratio)	Boys	0.85 ± 0.06	0.85 ± 0.06	0.662
	Girls	0.82 ± 0.08	0.80 ± 0.07	0.059

** *p* ≤ 0.01.

**Table 5:** Anthropometric characteristics of adolescents according to geographic regions (ANOVA).

		Northern	Central	Coastal	*p*	Post-Hoc
Arm Span (cm)	Boys	169.17 ± 11.25	169.23 ± 10.17	168.36 ± 9.43	0.892	/
	Girls	162.42 ± 6.97	164.37 ± 7.49	164.68 ± 7.65	0.376	/
Body Height (cm)	Boys	168.27 ± 9.05	169.67 ± 8.98	169.42 ± 8.35	0.646	/
	Girls	162.88 ± 5.30	166.33 ± 6.66	164.06 ± 6.99	0.009 **	Ce > N
Body Mass (kg)	Boys	65.07 ± 13.36	59.13 ± 13.41	62.89 ± 12.75	0.016 *	N > Ce
	Girls	56.17 ± 9.81	54.85 ± 9.23	56.53 ± 9.12	0.494	/
BMI	Boys	22.97 ± 4.36	20.39 ± 3.55	21.91 ± 3.40	0.000 **	N > Ce
	Girls	21.15 ± 3.49	19.78 ± 2.89	20.93 ± 2.77	0.011 *	/
Waist Circumference (cm)	Boys	75.33 ± 9.79	73.74 ± 9.29	71.94 ± 8.12	0.258	/
	Girls	67.02 ± 5.62	68.43 ± 7.02	66.34 ± 7.86	0.175	/
Hip Circumference (cm)	Boys	88.97 ± 9.35	86.12 ± 9.80	87.58 ± 7.89	0.172	/
	Girls	84.96 ± 6.88	83.88 ± 9.06	84.79 ± 9.67	0.735	/
WHR (Waist-to-Hip Ratio)	Boys	0.85 ± 0.06	0.86 ± 0.06	0.82 ± 0.06	0.003 **	Ce > Co
	Girls	0.79 ± 0.05	0.82 ± 0.08	0.79 ± 0.08	0.007 **	Ce > N Ce > Co

Legend: N—Northern; Ce—Central; Co—Coastal; *p*—significance level. * *p* ≤ 0.05. ** *p* ≤ 0.01.

## Data Availability

We don’t have the original data on any website. However, if needed, we will provide it to you.
